# Lifestyle and diabetes mellitus in cats and dogs

**DOI:** 10.1186/1751-0147-57-S1-K4

**Published:** 2015-09-25

**Authors:** Charlotte Bjørnvad

**Affiliations:** 1 Department of Veterinary Clinical and Animal Sciences, University of Copenhagen, Copenhagen, Denmark

## 

In westernised countries, obesity is the most commonly diagnosed nutritional disorder in dogs and cats, with an estimated prevalence of between 22% and 44% in investigated populations. In humans, it is well established that obesity predisposes to lifestyle-related diseases such as type 2 diabetes mellitus and atherosclerosis. Dogs and cats seem less prone to developing atherosclerosis compared with humans, while diabetes is a relatively common endocrine disease in both species. The high prevalence of obesity in pet dogs and cats is believed to be multifactorial, often relating to lifestyle and the fact that pets have moved from being working animals to becoming close family members. Influencing factors include neutering, inactivity, genetic predisposition as well as factors such as interaction with owners through feeding/treats and the owners lacking recognition of their pets being overweight. As in humans, obesity causes insulin resistance and impaired glucose metabolism in cats and dogs. In cats, this may progress into feline diabetes because long-term elevated insulin demands together with obesity-related metabolic alterations result in destruction of the beta cells – through mechanisms similar to development of human type 2 diabetes mellitus. In dogs, the pancreas seems to better cope with increasing demands for insulin production and type 2-like diabetes seldom develops. Obesity may however, still predispose for diabetes in dogs. A possible explanation could be that obese dogs are at risk of developing pancreatitis as a frequent consequence of pancreatitis in dogs is inflammation-mediated destruction of the beta cells.

